# IL7-IL12 Engineered Mesenchymal Stem Cells (MSCs) Improve A CAR T Cell Attack Against Colorectal Cancer Cells

**DOI:** 10.3390/cells9040873

**Published:** 2020-04-03

**Authors:** Andreas A. Hombach, Ulf Geumann, Christine Günther, Felix G. Hermann, Hinrich Abken

**Affiliations:** 1 Center for Molecular Medicine Cologne, Tumor Genetics, University of Cologne, and Department I Internal Medicine, University Hospital Cologne, D-50931 Cologne, Germany; andreas.hombach@uk-koeln.de; 2 Apceth Biopharma GmbH, D-81377 Munich, Germany; u.geumann@apceth.com (U.G.); f.hermann@apceth.com (F.G.H.); 3 Department for Genetic Immunotherapy, Regensburg Center for Interventional Immunology, and University Hospital Regensburg, D-93053 Regensburg, Germany

**Keywords:** CAR T cells, MSC, adoptive cell therapy, genetic immunotherapy, IL7, IL12

## Abstract

Chimeric antigen receptor (CAR) redirected T cells are efficacious in the treatment of leukemia/lymphoma, however, showed less capacities in eliminating solid tumors which is thought to be partly due to the lack of cytokine support in the tumor lesion. In order to deliver supportive cytokines, we took advantage of the inherent ability of mesenchymal stem cells (MSCs) to actively migrate to tumor sites and engineered MSCs to release both IL7 and IL12 to promote homeostatic expansion and Th1 polarization. There is a mutual interaction between engineered MSCs and CAR T cells; in presence of CAR T cell released IFN-γ and TNF-α, chronic inflammatory Th2 MSCs shifted towards a Th17/Th1 pattern with IL2 and IL15 release that mutually activated CAR T cells with extended persistence, amplification, killing and protection from activation induced cell death. MSCs releasing IL7 and IL12 were superior over non-modified MSCs in supporting the CAR T cell response and improved the anti-tumor attack in a transplant tumor model. Data demonstrate the first use of genetically modified MSCs as vehicles to deliver immuno-modulatory proteins to the tumor tissue in order to improve the efficacy of CAR T cells in the treatment of solid malignancies.

## 1. Introduction

Adoptive cell therapy with chimeric antigen receptor (CAR) modified T cells induces lasting remissions of B cell leukemia and lymphomas [1,2], however, the efficacy of CAR T cell therapy against solid tumors is so far poor and T cells rapidly enter exhaustion [3]. Tremendous efforts are currently made to prolong the CAR T cell response in the hostile tumor tissue, particularly, the chronic inflammatory environment mediates T cell repression and exhaustion. To overcome the situation, we reason to shift the cytokine environment towards an acute immune response in order to sustain and prolong a CAR T cell response which will finally result in an improved control of solid tumor lesions.

In order to deliver biologically active proteins to the tumor tissue, we thought to use mesenchymal stem cells (MSCs) as vehicles due to several reasons; MSCs are major components of the tumor stroma of different tissue origin [4] and are key players in orchestrating the tumor bed with respect to the cellular composition and regulation. This is particularly due to the tremendous plasticity of MSCs to differentiate into different types of stromal cells [5] and their capability to secrete a variety of chemokines and cytokines [6,7]. In contrast to their physiological function in wound healing and tissue repair [8], in cancer tissues MSCs initiate and sustain an inflammatory milieu that promotes tumor progression [9]. This process is moreover sustained by cancer cells that reprogram tumor resident MSCs to release a variety of factors that sustain cancer cell growth and invasion [10]. Moreover, MSCs exhibit an inherent ability to home towards tumors of various tissue origin including carcinomas of breast, lung, pancreas, and ovary. The tumor homing capacity was used to deliver therapeutic products to the tumor site by applying genetically engineered MSCs [11,12,13,14,15,16,17]. In particular, the capacity to actively migrate into the tumor tissue and the ease to engineer the cells by viral vectors makes MSCs attractive for delivering transgenic immune modulators in order to shift the tumor bed from an inhibitory to an immune stimulatory environment [18]. For therapeutic purposes, human MSCs moreover have the advantage that the cells are invisible to allo-recognition which allows using these cells as an allogeneic “off-the-shelf” cell product.

We addressed whether bone marrow derived MSCs sustain a CAR T cell attack against colorectal cancer. MSCs were engineered to release both IL7 and IL12 in order to shift the chronic inflammatory profile in the tumor tissue into a more favorite one for an acute CAR T cell response. IL7 and IL12 modified MSCs (termed apceth-301) improved the CAR T cell activation as judged by cytokine production and cytolytic activities, increased CAR T cell amplification, and decreased activation induced cell death (AICD); the elimination of adenocarcinoma cells by CAR T cells was improved in a xenograft mouse model. To the best of our knowledge, this is the first report to use genetically modified MSCs to modulate the cytokine milieu in the tumor stroma in order to sustain a CAR T cell attack.

## 2. Materials and Methods

### 2.1. Cell lines and Reagents

HEK293T cells are human embryonic kidney cells that express the SV40 large T antigen [19]. The colon carcinoma cell lines Colo320 (ATCC CCL 220.1) and LS174T (ATCC CL-188) were obtained from ATCC, Rockville, MD, USA. The anti-CD3 monoclonal antibody (mAb) OKT3, anti-CD28 mAb 15E8 and CAR-specific anti-idiotypic mAb BW2064/36 were purified by affinity chromatography from OKT3 hybridoma (ATCC CRL 8001), 15E8 hybridoma (kindly provided by Dr. R. van Lier, Red Cross Central Blood Bank, Amsterdam, The Netherlands), and BW2064/36 [20] hybridoma supernatants, respectively. The PE-conjugated F(ab’)_2_ goat anti-human IgG antibody was purchased from Southern Biotechnology. Fluorochrome-conjugated anti-CD3 mAb was purchased from Miltenyi Biotec (Bergisch Gladbach, Germany). Respective fluorochrome-conjugated isotype controls were purchased from BD Biosciences (San Diego, CA, USA). Matched antibody pairs for capture and detection of human IFN-γ were purchased from BD Biosciences. Recombinant IL2 was obtained from Endogen (Woburn, MA, USA). Immunofluorescence was analyzed using a FACS-Canto^TM^ cytofluorometer equipped with the Diva software (Version 6.0, Becton Dickinson, Mountain View, CA, USA). 

### 2.2. Preparation of Human T Cells

Peripheral blood lymphocytes were obtained from healthy donors by Ficoll density centrifugation. T cells were initially activated by OKT3 and 15E8 mAbs (100 ng/mL each) and IL2 (500 U/mL) and further cultivated in the presence of IL2 (500 U/mL). 

### 2.3. Isolation and Generation of Human Mesenchymal Stem Cells (MSC)

MSC were isolated from bone marrow of healthy donors. Bone marrow was seeded into 5-layer culture stacks (Corning) and cultivated in apceth’s proprietary xeno-free Medium Bio-1. Once MSC colonies became visible, MSC were harvested and cryopreserved (representing the MSC stock). Cryopreservation medium consisted of human serum albumin (Baxter), Hydroxyethyl starch (Fresenius Kabi, Bad Homburg, Germany), and 10% (*v*/*v*) DMSO. Transduced and native MSC batches were obtained from these stocks. Cryopreserved MSC stocks were taken into culture in Bio-1 medium at a density of 1000 to 5000 cells/cm^2^. After 3–5 days MSCs were harvested for transduction with a retroviral vector encoding human IL7 and IL12 or IL7 alone. MSCs were mixed with viral vectors at a MOI between 2 and 15 and seeded in 5-layer stacks. Three days after transduction, medium was removed and replaced by Bio-1 medium containing puromycin (3 µg/mL). Cells were selected for 3–5 days and further cultivated in standard Bio-1 Medium until cryopreservation. MSCs underwent 3–5 passages before being cryopreserved.

### 2.4. Generation of γ-Retroviral Vector to Engineer MSC

The used retroviral vectors are based on the retroviral SIN-vector pSERS11 [21]. Cloning was performed by the “In-Fusion recombination Kit” (Clonetech, Mountain View, CA, USA) which allows cloning by recombination. From the parental vector, a deletion was introduced between the 3′ untranslated region and the wPRE element to remove the internal promoter and original transgene. In the next step the human EFS promoter was introduced. Additionally, an encephalomyocarditis virus internal ribosome entry site followed by the puromycin resistance gene pac (puromycin N-acetyltransferase) was introduced down-stream of the promoter. To generate the IL7/IL12 construct, the human IL7 and IL12 genes, separated by a P2A site, were introduced between the promoter and the IRES site. To generate the IL7 construct the human IL7 was introduced between the promoter and the IRES site. Schematic vector maps are shown in Supplementary Figure S1. Vectors were generated by transient transfection of 293T cells as previously described [22].

### 2.5. Chimeric Antigen Receptors (CARs)

Engineering of CARs with specificity for the carcinoembryonic antigen (CEA) with the modified CD28-CD3ζ signalling domain [23] as well as the retroviral modification of T cells were previously described in detail [20,24,25].

### 2.6. T Cell Modification

Human peripheral blood T cells were retrovirally engineered for CAR expression [26]. T cells were stimulated and transduced on day 2 or 3 by γ-retrovirus containing supernatants or by co-culture with virus producing 293T cells as described [27]. Retroviruses were produced by 293T cells upon transient transfection with the DNA of the GALV encoding and the gag/pol encoding helper plasmid, and the plasmid encoding the respective CAR. CAR expression was monitored by flow cytometry using an antibody against the common extracellular IgG1 Fc domain.

### 2.7. Flow Cytometry

For flow cytometric analysis, CAR T cells were stained with fluorochrome-labeled antibodies specific for human IgG1 and CD3, respectively, and recorded by a FACSCanto II flow cytometer equipped with the FACSDiva software (Version 6.0, BD Bioscience, San Jose, CA, USA). MSC characterization is reported in Supplementary Materials and Methods.

### 2.8. Activation of CAR T Cells

CAR T cells (2.5 × 10^4^ cells/well) were co-cultivated for 48 h in 96-well round bottom plates with tumor cells (each 2.5–5 × 10^4^ cells/well) and non-modified or cytokine-modified MSCs (5 × 10^3^ cells/well). Specific cytotoxicity of CAR T cells against antigen-positive target cells was monitored by an XTT based colorimetric assay [28] using the “Cell Proliferation Kit II” (Roche Diagnostics, Mannheim, Germany). Viability of tumor cells was calculated as mean values of six wells containing only tumor cells subtracted by the mean background level of wells containing medium only. Non-specific formation of formazane due to the presence of T cells was determined from triplicate wells containing T cells in the same number as in the corresponding experimental wells. The number of viable tumor cells in experimental wells was calculated as follows: viability (%) = (OD (experimental wells–corresponding number of T cells))/(OD (tumor cells + MSC without T cells)–OD (medium)) × 100. Cytotoxicity (%) was defined as 100–viability (%). In another set of experiments, 96-well plates were coated with 4 µg/mL of the CAR specific anti-idiotypic mAb BW2064/36 or PBS for control. CAR T cells were CFSE (Thermo Fisher Scientific, Meerbusch, Germany) labeled according to manufacturer’s recommendations and co-cultivated (2.5 × 10^4^/well) with unmodified or cytokine modified MSCs (5 × 10^3^–2 × 10^4^/well) for 5 days. Cells were recovered and stained with anti-human IgG-PE and 7-AAD (Sigma-Aldrich, St. Louis, MO, USA) to identify live and dead CAR T cells and the number of cycling cells was determined by flow cytometry.

### 2.9. ELISA

IFN-γ and IL10 in culture supernatants were monitored by ELISA by binding to the solid phase anti-IFN-γ and IL10 capture antibody (each 1 µg/mL), respectively, and detection by the biotinylated anti-IFN-γ or anti-IL10 detection antibody (0.5 µg/mL), respectively. The reaction product was visualized by a peroxidase-streptavidin conjugate (1:10,000) and ABTS (Roche Diagnostics, Basel, Switzerland).

### 2.10. Multiplex Immunoassay

Secretion of cytokines and chemokines by MSCs and CAR T cells co-cultivated with MSCs and tumor cells was analyzed by a 43-parameter multiplex immunoassay (ProcartaPlex^®^; eBioscience, Frankfurt am Main, Germany), according to the manufacturer’s recommendations. Briefly, MSCs (25,000 cells/mL) were cultivated for 72 h in presence or absence of exogenous IFN-γ (10 ng/mL) and TNF-α (10 ng/mL). CAR T cells were co-cultivated with CEA^+^ tumor cells and non-modified or cytokine modified MSCs. The assays were performed in quadruples and supernatants of two wells were pooled and subjected to multiplex analysis. Data are expressed as means of duplicates.

### 2.11. CAR T Cell Mediated Suppression of Tumor Growth

NSG mice (Charles River, Sulzfeld, Germany) (5–12 animals/group) were subcutaneously inoculated with LS174T colorectal cancer cells (2.5 × 10^6^ cells/animal), CAR T cells (2 × 10^6^ cells/animal) and non-modified or cytokine modified MSCs (4 × 10^5^ cells/animal). T cells without CAR served as control. Tumor volumes were recorded every 2–3 days. When tumors reached a volume of > 1500 mm^3^ mice were sacrificed. Survival and tumor-free survival of mice were recorded. Experiments were approved by the local ethics committee.

### 2.12. Statistics

Experimental results from independent representative experiments are reported as mean values ± standard deviation (SD). Significance analyses was performed by the two-sided Student’s T test and log rank test using Microsoft Excel and Graphpad Prism, respectively.

## 3. Results

### 3.1. CAR T Cells Modulate the Activity of MSCs

**Figure 1:**
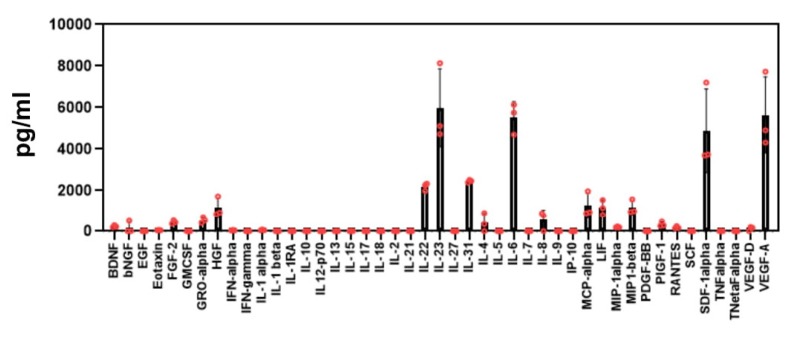
Mesenchymal stem cells (MSCs) from different donors secrete a similar pattern of cytokines and growth factors. Bone marrow derived MSCs (25,000/mL) of three donors were cultivated for 72 h. Supernatants were collected and subjected in duplicates to 43-parameter multiplex analysis as described in Materials and Methods. Data were grouped and mean values ± SD were determined.

Due to their tumor homing capacity tissues MSCs, with or without genetic engineering, are potential vehicles to deliver transgenic cell products that therapeutically modify the tumor tissue and improve an anti-tumor CAR T cell response. We isolated MSCs from the bone marrow of healthy donors and defined the cells according to the International Society for Cellular Therapy specifications [29] (Figure S2). MSCs were characterized to express CD73, CD90, and CD105 and to lack CD34 and CD45. The procedure of MSC preparation and ex vivo amplification was robust and reproducible since bone marrow derived MSCs from three different donors secreted a similar pattern of cytokines and growth factors with only minor quantitative differences into the supernatant as revealed by 43-parameter multiplex analysis (Figure 1).

**Figure 2:**
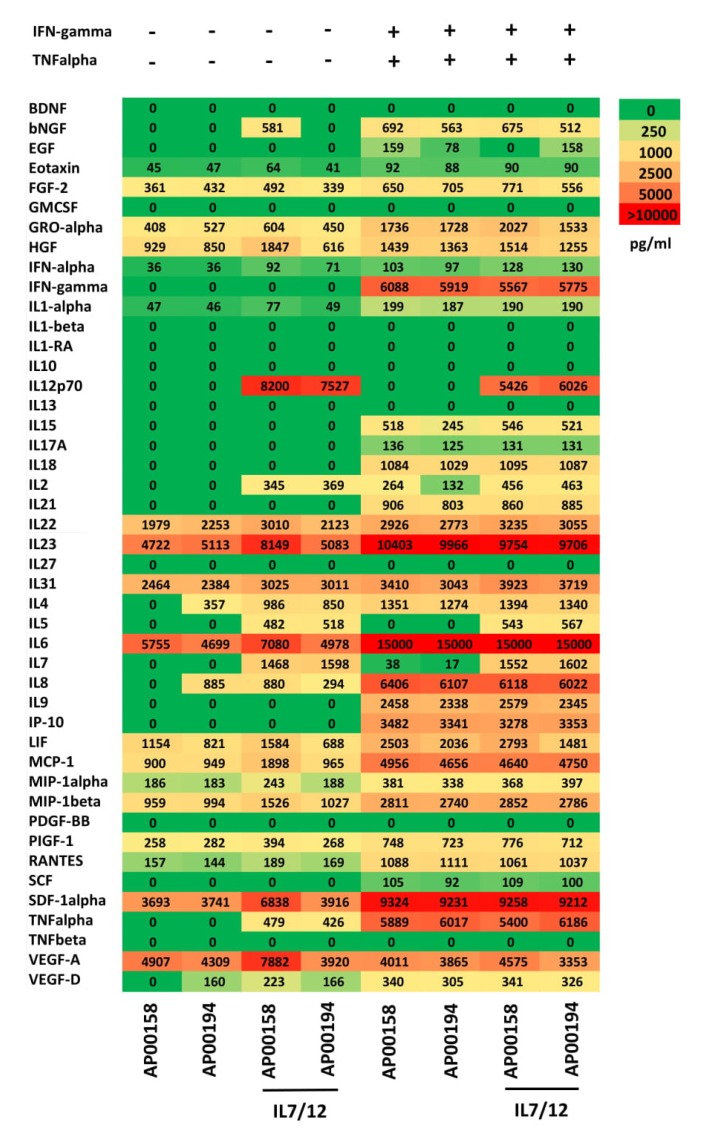
Non-modified MSCs and IL7/IL12 engineered MSCs have a similar pattern of secreted cytokines that is altered in presence of TNF-α and IFN-γ. MSCs of two donors were transduced to secrete IL7 and IL12, respectively, and cultivated for 72 h (25,000/mL) in presence and absence of added TNF-α (10 ng/mL) and IFN-γ (10 ng/mL), respectively. Supernatants were collected and subjected in duplicates to 43-parameter multiplex analysis as described in Materials and Methods.

In order to alter the cytokine profile in the tumor tissue we modified the MSCs by genetic engineering to release IL7, IL12, and both IL7 and IL12, respectively (Figure 2). A representative flow cytometric analysis of genetically modified MSCs is shown in Figure S2B. The transduction procedure itself did not impair the ability of MSCs to differentiate into osteoblasts and adipocytes as shown in Figure S2C. Moreover, genetically modified MSCs with release of transgenic IL7 and IL12 secreted basically a similar pattern of cytokines as the parental MSCs (Figure 2).

When in proximity to activated CAR T cells, MSCs are facing a panel of pro-inflammatory cytokines like IFN-γ and TNF-α secreted by CAR T cells. We asked whether the cytokine secretion pattern of MSCs will be altered by T cell secreted pro-inflammatory cytokines. MSCs and IL7/12 engineered MSCs from two donors were cultured in the presence of added IFN-γ and TNF-α. As summarized in Figure 2, IFN-γ and TNF-α induced in MSCs up-regulation of secretion of several pro-inflammatory cytokines including IL6 and IL23 indicating an enforced inflammatory reaction. This is not due to transgenic IL7 and IL12 since unmodified MSCs altered the pattern of cytokine secretion in a similar fashion.

**Figure 3:**
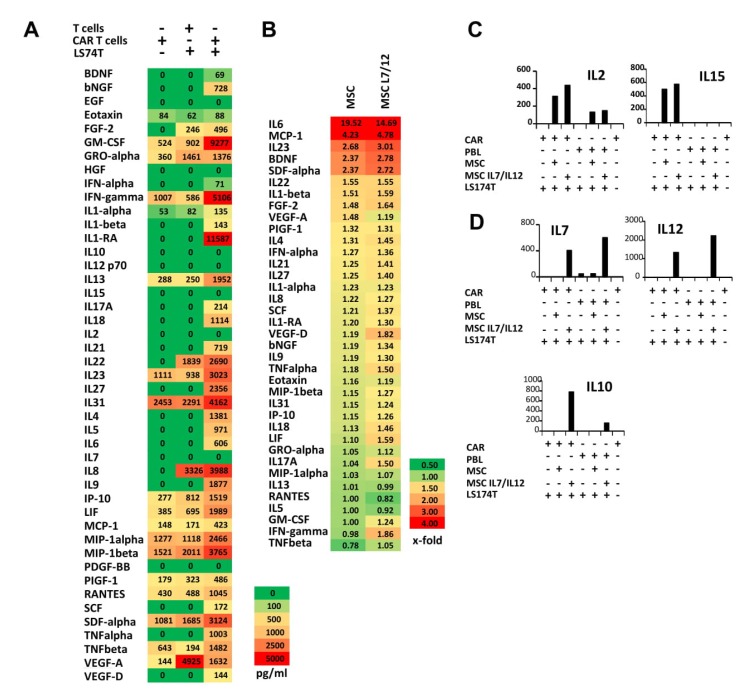
Chimeric antigen receptor (CAR) triggered cytokine secretion pattern of CAR T cells is specifically altered by MSCs. (**A**) Anti-carcinoembryonic antigen (CEA) CAR T cells were co-cultivated in 96-well plates (2.5 × 10^4^ cells/well) in quadruples with LS174T tumor cells (2.5 × 10^4^ cells/well), respectively. After 48 h supernatants were recovered and supernatants of 2 adjacent wells were pooled and subjected to 43-parameter multiplex analysis as described in Materials and Methods. Mean values of duplicates were determined and data were displayed as heat map. (**B**–**D**) Anti-CEA CAR T cells were co-cultivated with LS174T cancer cells as described above in presence of wt or IL7/IL12 modified MSCs (5 × 10^3^ cells/well) and subjected to 43-parameter multiplex analysis as described above. The number of secreted cytokines and growth factors was determined, and x-fold secretion compared to release of cytokines in presence of CAR T cells and tumor cells alone was calculated and presented as heat map (**B**). Cytokine secretion was dependent on the presence of MSC (**C**) or IL7/12 producing MSCs (**D**), data indicate mean values of duplicates.

To address how MSCs impact a tumor specific CAR T cell attack, we engineered T cells with a second generation CD28Δ-ζ CAR that is deficient in IL2 secretion due to a mutation in the lck binding domain of CD28 [30]. Upon transduction, the CAR was expressed in about 30–40% of T cells (Figure S3). Anti-CEA CAR T cells were co-cultured with CEA^+^ LS174T cells for 48 h and the supernatants were tested for cytokines and growth factors by 43-parameter multiplex analysis (Figure 3). Upon engagement of tumor cells, CAR T cells secreted high amounts of pro-inflammatory cytokines like IFN-γ, IL23, and TNF-α (Figure 3A). Adding MSCs resulted in enhanced the production of pro-inflammatory cytokines during a CAR T cell attack (Figure 3B) likely due to the activation of MSCs through CAR T cell derived TNF-α and IFN-γ (cf. Figure 2). In presence of IFN-γ and TNF-α, MSCs released IL2 and IL15 (Figure 3C) that, on the other hand, mutually activated CAR T cells indicated by high IL6 and IL23 release which enforced a pro-inflammatory Th17 response. We concluded that CAR T cells and MSCs, with or without transgenic IL7 and IL12, cooperate in up-regulating Th17 cytokines. Increased IL7 and IL12 in presence of transduced MSCs was accompanied by IL10 release during antigen-specific activation of CAR T cells (Figure 3D). This is in accordance with a previous report that IL12 enhanced IL10 secretion by T cells [31]. Taken together, CAR T cells and MSCs can mutually cooperate in enhancing a Th17 inflammatory anti-tumor response; IL7 and IL12 producing MSCs shift the T cell response to a Th1 like anti-tumor response.

### 3.2. MSCs Improved Antigen-Specific Amplification and Survival of CAR T Cells

**Figure 4:**
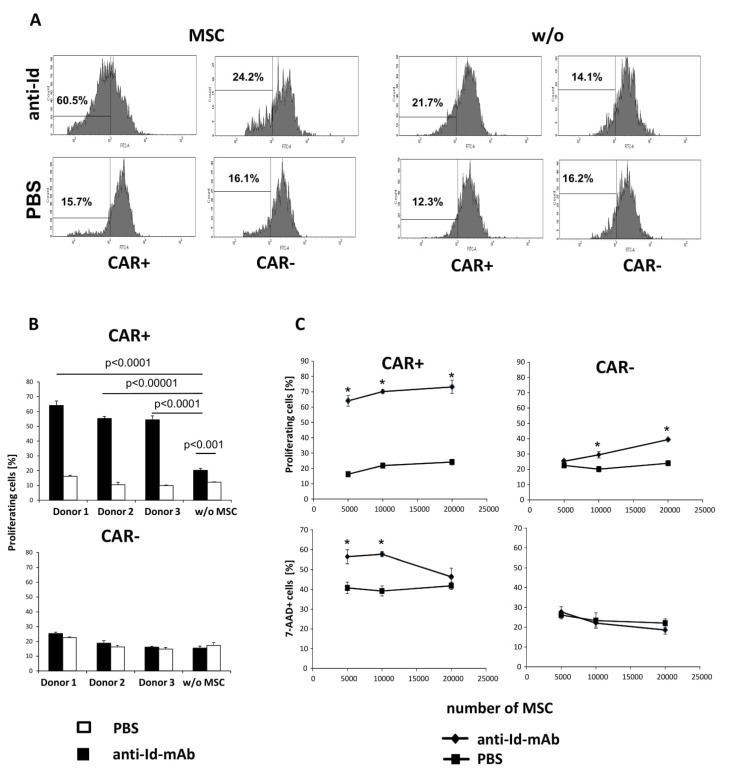
MSCs modulate antigen driven CAR T cell amplification. Anti-CEA CAR T cells were CFSE labeled and co-cultivated (2.5 × 10^4^ cells/well) together with MSCs in 96-well plates that were coated with the CAR specific anti-idiotypic mAb BW2064 (4 µg/mL) or PBS for control. After 5 days cells were recovered, CAR T cells stained with an anti-IgG-PE antibody to detect the CAR and analyzed by flow cytometry. Dead cells were identified by 7-AAD staining (1 µg/mL) and the number of proliferating cells was determined by CFSE dilution. (**A**, **B**) CAR T cell proliferation in presence of MSCs (each 5000 cells/well) of three different donors. (**A**) Histograms of a typical experiment. (**B**) Summary of CAR T cell proliferation in presence of MSC. (**C**) CAR T cell proliferation and cell death in presence of increasing numbers of MSCs (5000–20,000 cells/well). Values represent the mean of replicates +/− standard deviation (SD). Significant differences were calculated by Student’s T test; * *p* < 0.01.

**Figure 5:**
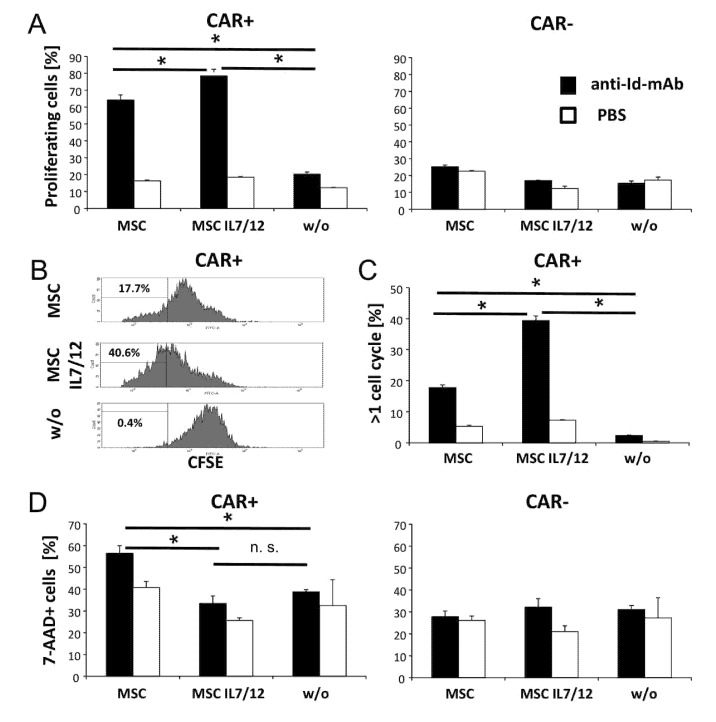
IL7 and IL12 secreting MSCs enhance CAR driven T cell proliferation and reduce activation induced cell death (AICD). Anti-CEA CAR T cells were CFSE labeled and co-cultivated (2.5 × 10^4^ cells/well) with or without non-modified or IL7/IL12-modified MSCs in 96-well plates (5000 cells/well) that were coated with the anti-idiotypic mAb BW2064 (4 µg/mL) for CAR engagement or with PBS for control. After 5 days cells were recovered, stained with an anti-IgG-PE antibody to detect the CAR and analyzed by flow cytometry. The number of proliferating T cells was determined by CFSE dilution, dead cells were identified by 7-AAD staining (1 µg/mL) and the number of proliferating cells was determined. (**A**) CAR T cell proliferation in presence or absence of MSCs; (b,c) number of rapidly cycling CAR T cells within the observation period; (**B**) flow cytometric histograms of a typical experiment after stimulation with the anti-idiotypic mAb, (**C**) summary of rapidly cycling CAR T cells. (**D**) AICD of CAR T cells in presence or absence of MSCs. Values represent the mean of replicates +/− standard deviation (SD). Significant differences were calculated by Student’s T test. *p* < 0.05, n.s., not significant.

By altering the cytokine secretion pattern, MSCs may impact proliferation and persistence of CAR T cells upon antigen engagement. MSCs were co-cultured with CFSE-labeled CAR T cells in the presence of the solid phase mAb BW2064 that is an anti-idiotypic mAb against the CAR binding domain and acts as surrogate antigen. MSCs increased amplification of CAR T cells when co-incubated at a ratio of 1:4 MSC:CAR T cells (Figure 4A); higher MSC numbers inhibited activation induced cell death (AICD) (Figure 4B). We concluded that added MSCs improve CAR driven T cell amplification and survival in vitro. The number of CAR T cells even more increased in the presence of IL7 and IL12 secreting MSCs (Figure 5A). This is due to increased numbers of cycling CAR T cells (Figure 5B) and a decreased frequency of AICD. AICD of CAR T cells was reduced even at low numbers of IL7/IL12 modified MSCs which was less the case in presence of non-modified MSCs (Figure 5C).

### 3.3. IL7 and IL12 Engineered MSCs Modulate the Cytotoxic CAR T Cell Attack

**Figure 6:**
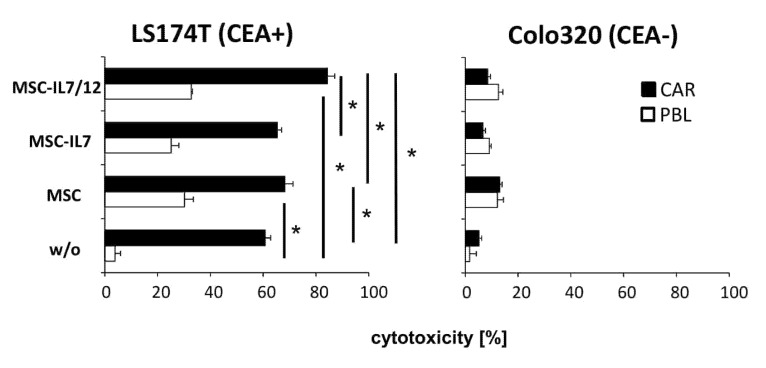
Cytokine engineered MSCs modulate a cytolytic CAR T cell attack. Anti-CEA CAR T cells and non-modified T cells for control (each 1.5 × 10^4^ cells/well) were co-cultivated for 48 h with CEA^+^ LS174T or CEA^−^ Colo320 tumor cells (each 2.5 × 10^4^ cells/well) and non-modified or IL7 and IL7/IL12 secreting MSCs (each 3 × 10^3^ cells/well) in 96-well round bottom plates. Viability of tumor cells was determined by a tetrazolium salt based XTT-assay. Cytolysis [%] was determined by recording the reduction in viability. Values represent the mean of replicates +/− standard deviation (SD). Significant differences were calculated by Student’s T test, * *p* < 0.05.

To address whether MSCs modulate the cytotoxic CAR T cell attack against antigen-positive tumor cells we co-cultivated anti-CEA CAR T cells with CEA^+^ LS174T and CEA^−^ Colo320 tumor cells in the presence of MSCs and recorded CAR mediated target cell lysis (Figure 6). CAR T cell mediated elimination of CEA^+^ target cells was increased in the presence of IL7 and IL12 releasing MSCs compared with non-modified MSCs. Increased target cell lysis was due to IL12 because IL7 releasing MSCs without IL12 did not enhance CAR mediated cytotoxicity. Notably, non-modified MSCs also enhanced the anti-tumor cell reactivity of CAR T cells and non-modified T cells.

### 3.4. IL7 and IL12 Secreting MSCs Sustain the Overall Anti-Tumor Response in a Transplant Tumor Model

**Figure 7:**
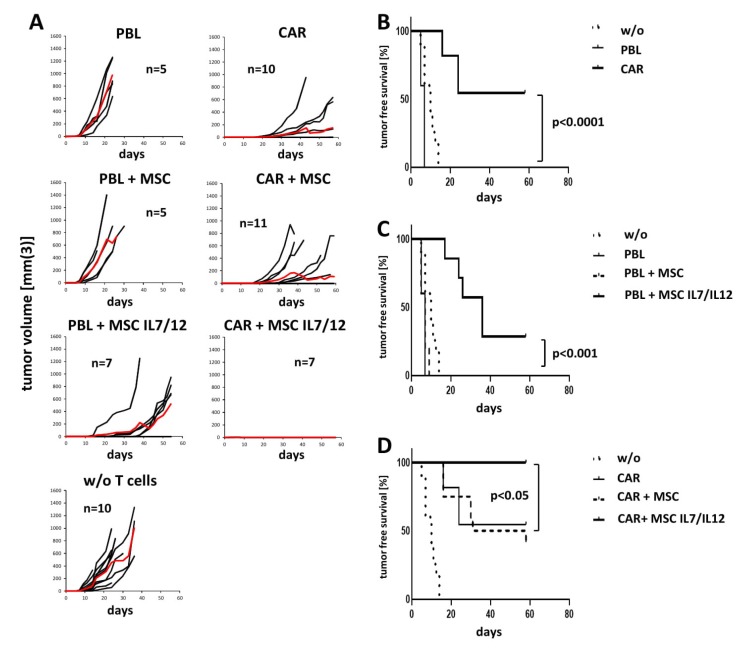
IL7 and IL12 secreting MSCs support an anti-tumor response in vivo. NSG mice (5–12 animals/group) were subcutaneously co-injected with LS174T tumor cells (2.5 × 10^6^/mouse), anti-CEA CAR T cells or non-modified T cells (2 × 10^6^/mouse) in presence or absence of non-modified or IL7 and IL12 secreting MSCs (4 × 10^5^/mouse). Tumor growth was monitored every 2–3 days. Onset of tumor growth was determined at tumor size >20 mm^3^. The experiment was terminated on day 58. (**A**) Tumor growth of individual animals; red line represents mean values. (b–d) Kaplan Meier plots of data with tumor free survival. (**B**) Comparison of groups with non-modified and CAR T cells; (**C**) comparison of groups with non-modified T cells in presence or absence of MSCs and IL7/IL12 secreting MSCs; (**D**) comparison of groups with CAR T cells in presence or absence of MSCs and IL7/IL12 secreting MSCs. Statistical analysis was determined by the Logrank-test.

Despite the high tumor tropism of engineered MSC [32], the majority of intravenously applied MSCs in the xenogenic mouse disappeared rapidly from the circulation during the lung passage [33] and those that persist in the circulation required a long time period for accumulation in the tumor tissue. We therefore addressed whether IL7/IL12 modified MSCs improve the CAR T cell anti-cancer cell attack by local application of MSCs and CAR T cells in a similar fashion as described previously by others [34,35,36,37]. We co-injected NSG mice with anti-CEA CAR T cells, IL7/IL12 modified MSCs, and CEA^+^ tumor cells. T cells without CAR and non-modified MSCs without IL7 and IL12 secretion served as controls. CAR T cells alone suppressed tumor formation and improved the survival (Figure 7A). T cells without CAR also took benefit from IL7 and IL12 secreting MSCs resulting in prolonged survival; this was not the case when co-inoculating non-modified MSCs (Figure 7B). The effect is due to MSC released IL7 and IL12 since non-modified MSCs had no impact on the anti-tumor activity of CAR T cells (Figure 7C).

## 4. Discussion

We here report on the use of bone marrow derived MSCs engineered with IL7 and IL12 to sustain a CAR T cell attack against colorectal cancer. The concept takes benefit of the unique capacity of MSCs to migrate to and to infiltrate into solid tumors [18] and, upon genetic modification, to produce and deliver transgenic cytokines to the tumor.

MSC engineering with a retroviral vector encoding the production and release of IL7 and IL12 was efficient and without altering the cell phenotype. The use of these cytokines is based on the rationale that IL7 promotes the homeostatic expansion and sustains memory cell function of T cells [38], both crucial for the success of CAR T cell therapy. IL12 induces a protective Th1 response and prevents Th2 polarization of T cells [39] and, moreover, attracts and activates an innate immune response to eliminate those cancer cells that are invisible to CAR T cells [40]. Both cytokines were not produced by CAR T cells or MSCs but impact the CAR T cell response. Screening of the panel of cytokines released during a CAR T cell anti-tumor cell attack in the presence of engineered MSCs revealed that the chronic inflammatory Th2 response of non-modified MSCs shifted towards a Th17/Th1 pattern that is supportive for a T cell anti-tumor response. In particular, there is a CAR T cell mediated pattern of cytokines that was up-regulated by non-modified and IL7/IL12 engineered MSCs. The release of anti-tumorigenic cytokines like IFN-γ, IL6, IL23, IL18, and TNF-α was enhanced in the presence of IL7/IL12 engineered MSCs; these cytokines will likely shift the pro-tumorigenic chronic inflammatory situation to a more anti-tumorigenic acute inflammation capable to sustain an anti-tumor response by CAR T cells and potentially other infiltrating adaptive and innate immune cells [41].

While MSCs are pivotal regulatory constituents of the tumor stroma by promoting tumor progression and invasion [4,42], our data revealed that there can be a mutual cross-talk between MSCs and anti-tumor T cells with substantial impact on the overall tumor control. The pattern of cytokines released by non-modified MSCs resembles a chronic inflammatory Th2 reaction capable to support tumor growth rather than a T cell anti-tumor attack. During a CAR T cell attack through released IFN-γ and TNF-α, the MSCs shift towards a more acute inflammatory response resulting in an improved anti-tumor reactivity and persistence of CAR T cells. While non-modified MSCs enhance a Th17 dominated pro-inflammatory immune response, IL7 and IL12 engineered MSCs shifted the CAR T cell response towards Th1 inflammation with extended CAR T cell persistence indicated by robust killing, T cell amplification, and protection from activation induced cell death. In a transplanted xenograft tumor model, IL7/IL12 engineered MSCs boosted the anti-tumor activity of CAR T cells while non-modified MSCs do not have the effect.

The impact of MSCs on immune regulatory functions of CAR T cells is complex and depends on the MSC to CAR T cell ratio. In the presence of low numbers of non-modified MSCs the CAR driven T cell proliferation is improved whereas the inhibition of AICD requires higher numbers of MSCs. The situation is different in the presence of IL7 and IL12 engineered MSCs due to the increased levels of acute pro-inflammatory cytokines that drive Th1 polarization. IL12 enhanced antigen-specific CAR T cell proliferation, reduced AICD and enhanced target cell lysis which was not observed in the presence of IL7 MSCs. The presence of IL12, however, induced also IL10 secretion in CAR T cells that may have double impact on an immune attack against cancer cells: antigen presentation of APC may be inhibited but on the other hand IL10 will modify cancer promoting chronic inflammation. Whereas the role of IL10 on cancer growth may be ambiguous [43,44] we expect an overall beneficial impact of IL7/12 engineered MSC on a CAR T cell attack.

Upon tumor infiltration, MSCs arrange themselves in close proximity with tumor cells and vasculature [45], making them ideal vehicles for the delivery of cytokines that are picked up by infiltrating immune cells including CAR T cells. There is an ongoing discussion whether the inherent MSCs homing and retention capabilities are sufficient for a lasting therapeutic effect; engineering MSCs with a truncated, non-signaling CAR was applied in order to increase tumor affinity [46] and to improve delivery of therapeutic proteins [47,48]. MSCs were modified for targeting by transduction with a targeting domain like a chimeric receptor targeting EGFRvIII [45,49] and GD2 [46]. MSCs were engineered with both to deliver TRAIL and to specifically recognize GD2 through a truncated anti-GD2 CAR in order to improve their retention in the targeted GD2 positive tumors like glioblastoma, sarcomas and neuroblastoma [46]. These modified MSCs mediate an improved anti-tumor response with a prolonged cell-to-cell interaction and thereby more effectively delivering TRAIL. The first step to use engineered MSCs to support a T cell anti-tumor response was most recently reported by Szoor et al. [50] who engineered MSCs to release a bispecific T cell engager and to provide CD80 and 4-1BB costimulation. The T cells themselves, however, were not modified in order to redirect their cytolytic activities in a pre-defined fashion. In that situation the MSCs needed to provide both costimuli CD80 and 4-1BB in order to induce T cells for antigen-dependent IL2 release and tumor cell killing. In our concept, however, the redirected T cells obtained co-stimulation by the CAR and were supported in prolonging their response through IL7/IL12 delivered by the MSCs.

Whereas modified MSCs clearly improved the anti-cancer cell activities of CAR T cells in vitro but also induces anti-tumor reactivity in CAR^−^ lymphocytes, the situation in vivo seems to be more complex. In a xenograft model, CAR T cells efficiently suppressed tumor growth in the presence of non-modified MSCs that could only slightly be improved in the presence of IL7/IL12 engineered MSCs. The same IL7/IL12 MSCs enhanced the allogeneic response of T cells independently of the CAR implying that IL7/IL12 engineered MSCs booster the specific T cell response and initiate antigen-independent anti-tumor activities. This may be due to high secretion of IL22 and IL23 by MSCs. These cytokines were recently described to recruit and activate CD56^+^ cells for an innate immune response [51,52,53] and were secreted independently of engineering IL7/IL12 secretion and may activate non-specific cytotoxicity against cancer cells that is further boosted by IL12 of engineered MSC. A recent report demonstrated a local effect of IL7/12 engineered MSC on tumor infiltrating cells as well as a systemic effect on circulating lymphocytes resulting in up-regulation of activation markers [37]. Accordingly, a CAR T cell attack and acquisition of antigen-independent cytotoxicity will synergistically benefit from both effects. Hereby, IL7 will improve persistence of CAR T cells and other cytotoxic lymphocytes in the periphery and the tumor tissue whereas IL12 will directly boost anti-tumor cytotoxicity as demonstrated in this report.

The acquisition of antigen-independent anti-tumor activities of CAR T cells is of particular relevance when eliminating tumors with a heterogeneous pattern of targetable antigens. This holds for the majority of solid tumor lesions that harbor cancer cells lacking the antigen and rendering those cancer cells invisible to CAR T cells which potentially give rise to tumor relapse after initially successful tumor reduction. The IL7/IL12 MSC and CAR T cell-based approach combines both strategies and delivers cytokines that sustain both a redirected antigen-specific and an antigen-independent immune cell response. MSCs engineered to release IL7 and IL12 as “of-the-shelf cell therapy additives” will help to establish a pro-inflammatory response that prolongs the T cell anti-tumor attack and successfully counteracts repression through the tumor environment.

## 5. Conclusions

MSCs engineered to release IL7 and IL12 shift the chronic inflammatory Th2 profile of the tumor microenvironment into a more favorite Th17/Th1 profile with an improved and prolonged anti-tumor CAR T cell response. Data demonstrate the use of genetically modified MSCs as vehicles to modulate the inflammatory tumor microenvironment and to establish a mutual stimulatory interaction between modified MSCs and CAR T cells during an anti-tumor attack.

## Supplementary Materials

The following are available online at https://www.mdpi.com/2073-4409/9/4/873/s1, Figure S1: Transgene cassette EFS_Il-7_P2A_IL-12A T2A_IL12b_IRES_pac, Figure S2: Characterization of IL7/IL12 transduced MSC, Figure S3: Expression of anti-CEA CAR in T cells from the peripheral blood, Table S1: Antibodies for characterization of MSCs.
