# Supplementary material for: IL7-IL12 Engineered Mesenchymal Stem Cells (MSCs) Improve A CAR T Cell Attack Against Colorectal Cancer Cells
Source: Cells. 2020 Apr 3;9(4):873. doi: 10.3390/cells9040873 (PMC7226757; doi:10.3390/cells9040873)
Supplement: Supplementary file 1 [file cells-09-00873-s001.pdf]

Supplementary Methods:

MSC surface flow cytometric analysis IL7/IL12 MSCs were cultivated for three to five days, harvested, split into three vials and stained with three antibody combinations: (1) isotype controls; (2) CD34, CD45, CD73, CD90, CD105; (3) CD3, CD14, CD19, CD41, CD235a. Antibodies are listed below (Supplementary Table 1). Flow cytometric analysis was performed with a MACSQuant® system (Miltenyi Biotec). Supplementary Figure 1 shows an excerpt of data.

MSC differentiation 60×10^4 native or IL7/IL12 transduced MSCs were seeded in each well of a 24-well plate. For adipogenic or osteogenic differentiation, MSCs were cultured for two to three weeks in StemMACS OsteoDiff medium (Miltenyi Biotech) or StemMACS AdipoDiff medium (Miltenyi Biotech), respectively, according to the manufacturer's instructions. After differentiation, cells were fixed with 10% formaldehyde and adipocytes were stained with Oil Red (Sigma Aldrich), while osteocytes were stained with Alzerin Red (Sigma Aldrich).

Table S1. Antibodies for characterization of MSCs.

Antibody	Manufacturer	Clone
CD3-PC7	Becton Dickinson	SK7 (Leu-4)
CD14-APC	eBioscience	61D3
CD19-APC-Vio770	Miltenyi Biotec	LT19
CD34-PC7	Becton Dickinson	8G12
CD41a-PE	Becton Dickinson	HIP8
CD45-FITC	Becton Dickinson	2D1
CD73-APC	Becton Dickinson	AD2
CD90-APC-Vio770	Miltenyi Biotec	DG3
CD105-PE	Becton Dickinson	266
Glycophorin A- PE (CD235a-PE)	R&D Systems	R10
IgG1-APC	Becton Dickinson	MOPC-21
IgG1-APC-Vio770	Miltenyi Biotec	IS5-21F5
IgG1-FITC	Becton Dickinson	MOPC-21
IgG1-PE	Becton Dickinson	MOPC-21
IgG1-PC7	Becton Dickinson	MOPC-21

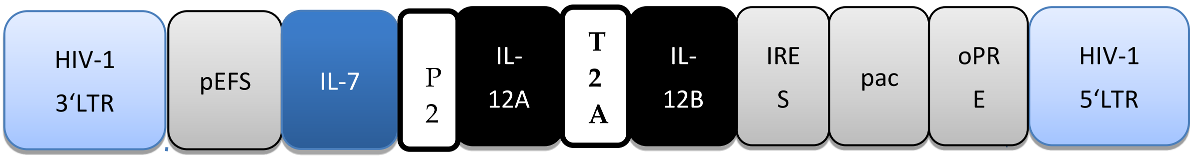

Figure S1. Transgene cassette EFS_Il-7_P2A_IL-12A T2A_IL12b_IRES_pac. LTR: HIV-1 derived long terminal repeats, pEFS: short intron-less form of elongation factor 1 alpha 1 (EF1a) promoter, Il-7: Interleukin-7 cDNA sequence, IL-12A: Interleukin 12 p35 subunit cDNA sequence, IL-12B: Interleukin p40 cDNA sequence, P2A and T2A: self cleaving elements, IRES: internal ribosomal entry site, pac: Puromycin-N-acetyltransferase, oPRE: woodchuck hepatitis post-transcriptional regulatory element.

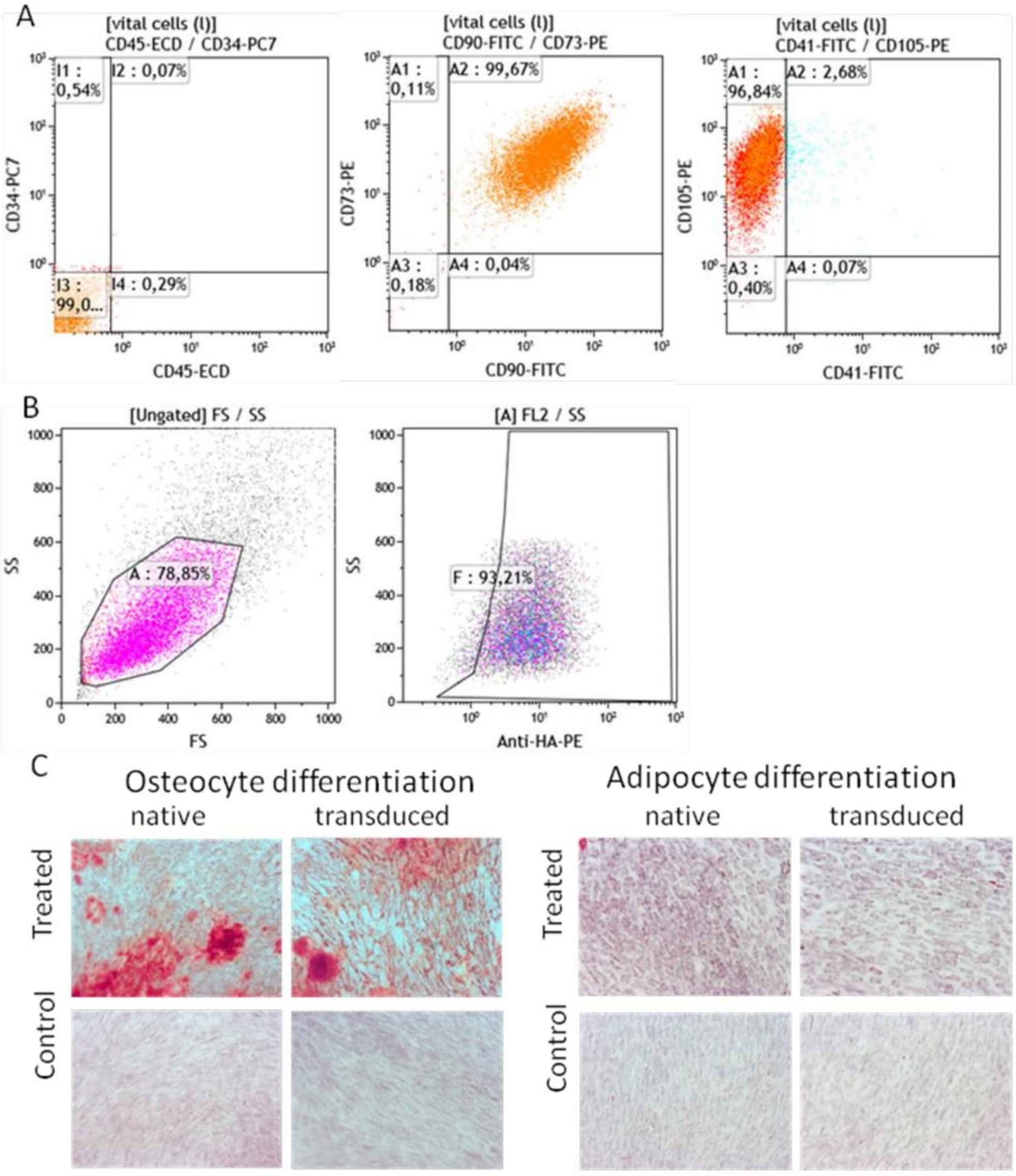

Figure S2. Characterization of IL7/IL12 transduced MSC. (A) Surface marker expression of IL7/IL12 transduced MSCs was analysed by flow cytometry as described in supplemental materials and methods (B) Intracellular FACS analysis of puromycin selected IL7/IL12 MSCs utilizing an anti-HA PE mAb for detection of recombinant cytokines (C) 60×104 native or IL7/IL12 transduced MSCs were seeded in each well of a 24-well plate. For adipogenic or osteogenic differentiation, MSCs were cultured for two to three weeks in StemMACS OsteoDiff medium (Miltenyi manufacturer's instructions. After differentiation, cells were fixed with 10% formaldehyde and adipocytes were stained with Oil Red (Sigma Aldrich), while osteocytes were stained with Alzerin Red (Sigma Aldrich).

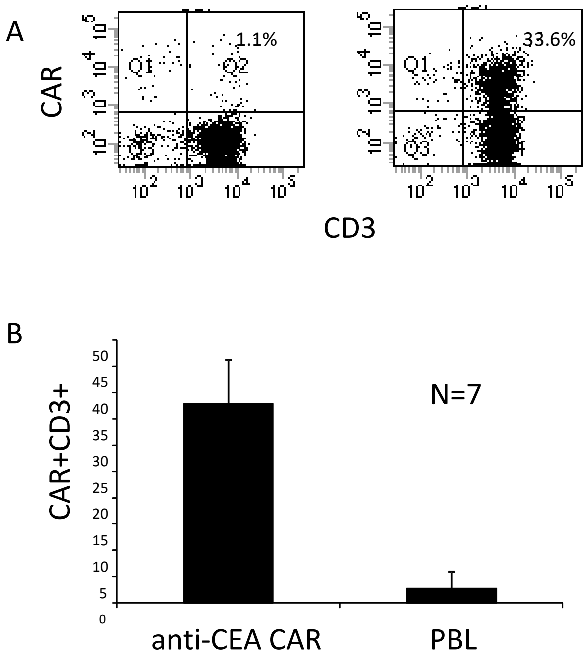

Figure S3. Expression of anti-CEA CAR in T cells from the peripheral blood. Density centrifugation isolated T cells from the peripheral blood of healthy donors were activated, cultivated and infected with retroviral vector encoding the anti-CEA CAR as described in Materials and Methods. T cells with CAR expression were identified by flow cytometry with anti-CD3 and anti-human IgG antibodies against the constant domain of the CAR. (**A**) Dot blot of a typical experiment; (**B**) mean values +/- SD of 7 randomly selected blood donors.
